# Genus *Caulophyllum*: An Overview of Chemistry and Bioactivity

**DOI:** 10.1155/2014/684508

**Published:** 2014-05-04

**Authors:** Yong-Gang Xia, Guo-Yu Li, Jun Liang, Bing-You Yang, Shao-Wa Lü, Hai-Xue Kuang

**Affiliations:** ^1^Key Laboratory of Chinese Materia Medica, Heilongjiang University of Chinese Medicine, Ministry of Education, Harbin 150040, China; ^2^Pharmaceutical College, Harbin Medical University, Harbin 150086, China

## Abstract

Recently, some promising advances have been achieved in understanding the chemistry, pharmacology, and action mechanisms of constituents from genus *Caulophyllum*. Despite this, there is to date no systematic review of those of genus *Caulophyllum*. This review covers naturally occurring alkaloids and saponins and those resulting from synthetic novel taspine derivatives. The paper further discussed several aspects of this genus, including pharmacological properties, mechanisms of action, pharmacokinetics, and cell membrane chromatography for activity screening. The aim of this paper is to provide a point of reference for pharmaceutical researchers to develop new drugs from constituents of *Caulophyllum* plants.

## 1. Introduction

*Caulophyllum* is a small genus of perennial herbs in the family Berberidaceae. The genus* Caulophyllum* is well known for its diversity and pharmacological uses in traditional medicine system since ancient times. All species in this genus are very similar [1].* C. robustum* is native to eastern Asia, especially in China, while* C. thalictroides* and* C. giganteum* are native to eastern North America. It is worth noting that nea

rly all phytochemical and pharmacological studies on this genus are focused on* C. thalictroides* and* C. robustum* due to their important medical functions [2].

The roots and rhizomes of* C. thalictroides* (L.) Michx. (blue cohosh) have been used traditionally by Native Americans for medicinal purposes [3]. The primary function of blue cohosh in many native communities of North America was to induce childbirth, ease the pain of labor, rectify delayed or irregular menstruation, and alleviate heavy bleeding and pain during menstruation [4]. Between 1882 and 1905, blue cohosh was listed in the United States Pharmacopoeia as a labor inducer [5] and sold as an herbal supplement that can aid in childbirth. Dietary supplements of blue cohosh are readily available throughout the USA over-the-counter and from Internet suppliers [6]. There is considerable concern about the safety of blue cohosh with reports of new born babies having heart attacks or strokes after the maternal consumption of blue cohosh to induce labor [7–9]. There is a heated discussion about using blue cohosh as dietary supplements for women [2].

*C. robustum* Maxim is well-known in* Hong Mao Qi* in Chinese, which grows widely throughout north-east, north-west, and south-west China. Its roots and rhizomes have been used as folk medicine to treat external injuries, irregular-menses, and stomach-ache due to its strong and wide biological activities [10]. Modern pharmacological studies have demonstrated that alkaloids and triterpence saponins are responsible for its major biological function as an anti-inflammatory [11], analgesic [12], antioxidant [13], antibacterial [11], antiacetylcholinesterase [14], and antitumor [15, 16]. Taspine, a lead compound in anticancer agent development [17, 18], was firstly screened to possess obvious effect on tumor angiogenesis and human epidermal growth factor receptor by using cell membrane chromatography from the* C. robustum* [19].

So it is very necessary to deeply explore* Caulophyllum* plants. In the past decades, some promising advances have been achieved in understanding the chemistry, pharmacology, and action mechanisms of constituents from genus* Caulophyllum*. From the opinion of safety of using dietary supplements of blue cohosh, a review dealing with quantitative methods of primary constituents of blue cohosh in dietary supplements has been published [2]. However, to date, there is no systematic review of chemistry, pharmacology, and action mechanisms of constituents from genus* Caulophyllum*.

In this review, the different structures of the alkaloids and saponins in genus* Caulophyllum* are described, including naturally occurring constituents and synthetical taspine derivatives. The present review highlighted the chemistry and pharmacological diversity and mechanism of action. The aim of this paper is to provide a point of reference on* Caulophyllum* plants for pharmaceutical researchers. Furthermore, various perspectives and existing problems for this genus are offered for consideration.

## 2. Phytochemistry

**Scheme 1:**
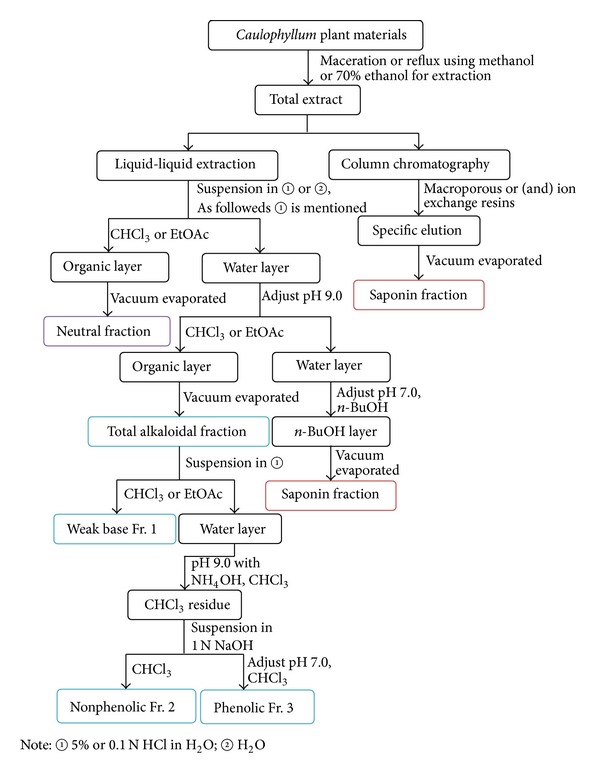
Summary of procedures for isolation of alkaloids and saponins from* Caulophyllum* plants.

Phytochemical research carried out on genus* Caulophyllum* led to the isolation of alkaloids and triterpence saponins and a few other classes of secondary metabolites. A comprehensive summary of structures and isolation methods of metabolites classified by structural types was given in present review.  Scheme 1 summarizes the procedures for crude isolation of alkaloids and triterpene saponins from genus* Caulophyllum*. The roots and rhizomes of* Caulophyllum* plants are extracted with methanol or 70% ethanol by maceration [13, 20] or reflux [21], and the combined extracts are concentrated in vacuo to dryness. Then two schemes are available for acquiring the alkaloid and saponin fractions, namely, liquid-liquid partition and liquid-solid column chromatography methods [21]. Liquid-liquid partition is commonly performed for crude isolation. In most cases, the residue is suspended in 5% or 0.1 N HCl in water and then partitioned with EtOAc or CHCl_3_ to remove neutral constituents. The aqueous layer was then removed, NH_4_OH was added to make it basic (pH 9), and the whole was extracted with EtOAc or CHCl_3_. The EtOAc or CHCl_3_ soluble part was evaporated to obtain the total alkaloidal fraction. Moreover, total alkaloidal fraction was able to further liquid-liquid partition to afford weak base (Fr. 1), nonphenolic alkaloids (Fr. 2), and phenolic alkaloids (Fr. 3) [13]. The H_2_O layer was neutralized with 5% HCl and extracted with* n*-butanol. The combined organic layers were evaporated to obtain total saponin fraction [20]. Column chromatography is also a popular method to enrich total alkaloids and saponins from* Caulophyllum* plants by choosing optimal macroporous or (and) ion exchange resins [13, 21, 22].

### 2.1. Alkaloids

**Figure 1:**
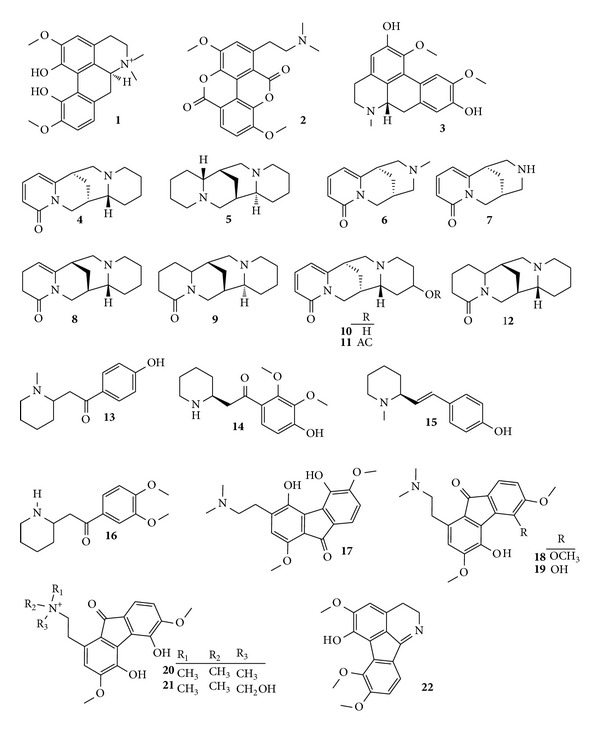
Chemical structures of alkaloids (**1**–**22**) from genus* Caulophyllum*.

With respect to alkaloid aspects of this genus, 22 molecules have been isolated and identified from genus* Caulophyllum*. Alkaloid compounds are very important bioactive constituents in genus* Caulophyllum*. Their chemical structures and sources can be seen in Figure 1 and Table 1. These compounds can be divided into several kinds of structural types. magnoflorine (**1**), taspine (**2**), and boldine (**3**) are contributed to aporphine alkaloids. Aporphine alkaloids have been shown to possess anticancer activity and there is evidence that this activity is exerted through induction of apoptosis, inhibiting cell proliferation and inhibiting DNA topoisomerase [23, 24]. Magnoflorine (**1**), a quaternary ammonium base, is isolated and detected with the biggest amounts among all the alkaloids isolated from genus* Caulophyllum*.** 1** was also isolated from the* n*-butanol fraction of blue cohosh due to its strong water-solubility, but it was not active in the rat embryo culture [25]. The molecular structure of** 2** is characterized by high symmetry.** 4**–**12** are typical quinolizidine alkaloids. Quinolizidine alkaloids have been reported to possess the obvious nematicidal activity [26].

In October 1999, a novel alkaloid, thalictroidine (**13**) with piperidine-acetophenone conjugate, was isolated from the rhizomes of* C. thalictroides* using an* in vitro* rat embryo culture method.** 13** was not teratogenic in the rat embryo culture at tested concentrations [25]. After nine years,** 13** was isolated again from* C. thalictroides*, together with** 14**–**16** [20].** 13–15**, piperidine-acetophenone conjugates, are rare in the plant kingdom.** 16** was only reported from* Boehmeria* genus [27] and is another example of such a type of compound from natural sources.

In April 2009, a distinct class of alkaloid, fluorenone alkaloid (caulophine,** 17**), was firstly reported from the radix of* C. robustum* using cell membrane chromatography as the screening method.** 17** was identified as 3-(2-(dimethylamino) ethyl)-4,5-dihydroxy-1,6-dimethoxy-9H-fluoren-9-one based on physicochemical and spectroscopic analyses.** 17** possessed antimyocardial ischemia activity by rat experiments. It is worth mentioning that a preparative high performance liquid chromatography method was developed for isolation, purification, and enrichment of caulophine (**17**) [28]. As follows, another four fluorenone alkaloids, caulophyllines A–D (**18**–**21**), and one dihydroazafluoranthene alkaloid, caulophylline E (**22**), were isolated from the roots of* C. robustum*.

Fluorenone alkaloid is a newly discovered alkaloid skeleton in natural products.** 17**–**21**, five new fluorenone alkaloids, were isolated from the same plant, suggesting that fluorenone type alkaloid is another kind of metabolites that existed in this genus* Caulophyllum*.** 22** is a novel and rare naturally occurred dihydroazafluoranthene alkaloid, there are no reports about dihydroazafluoranthene alkaloid isolated from natural products except its novel core skeleton first isolated from coal tar [24].** 22** has the isoquinoline fragment, which is possible to be the conceivable precursor of different substituted fluorenone alkaloids. A hypothetical biosynthetic pathway for** 20** was proposed starting from** 22**, which undergoes a sequential nitrogen-related double bond reduction, oxidation, ring-opening, N-methylation, and demethoxy process [13].

### 2.2. Triterpene Saponins

*Caulophyllum* triterpenes generally constitute the main class of secondary metabolites in the genus* Caulophyllum* amounting to up to 7.46% of the total dry weight in root and rhizome [29]. Until now, 32 caulophyllsaponins were isolated and identified by chemical and detailed spectroscopic analysis (Table 2). These saponins generally bear one (monodesmosidic) or two (bidesmosidic) carbohydrate chains that are directly attached to the hydroxyl groups in position C-3 for monodesmosidic saponins and to positions C-3 and C-28 in the case of the bidesmosidic saponins.** 25**,** 28**,** 29**,** 33**,** 34, 37**–**39,** and** 41**–**44** are bidesmosidic triterpenoid saponins with two sugar chains at C-3 and C-28. Others were found to only have one sugar chain at C-3 or C-28.

**Figure 2:**
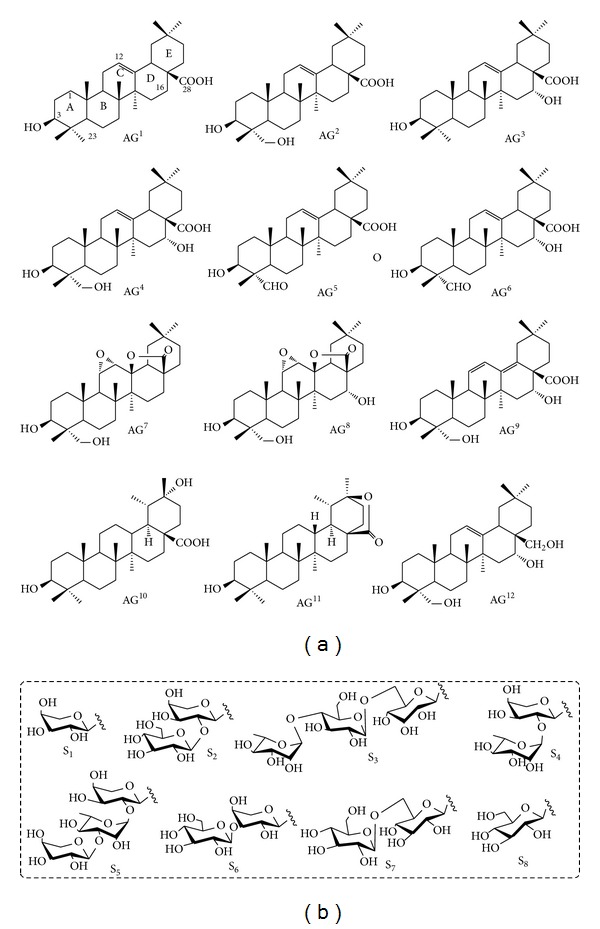
(a) Chemical structures of aglycones (AG) of saponins; (b) linkage mode of sugar moieties of saponins from genus* Caulophyllum*.

**Scheme 2:**
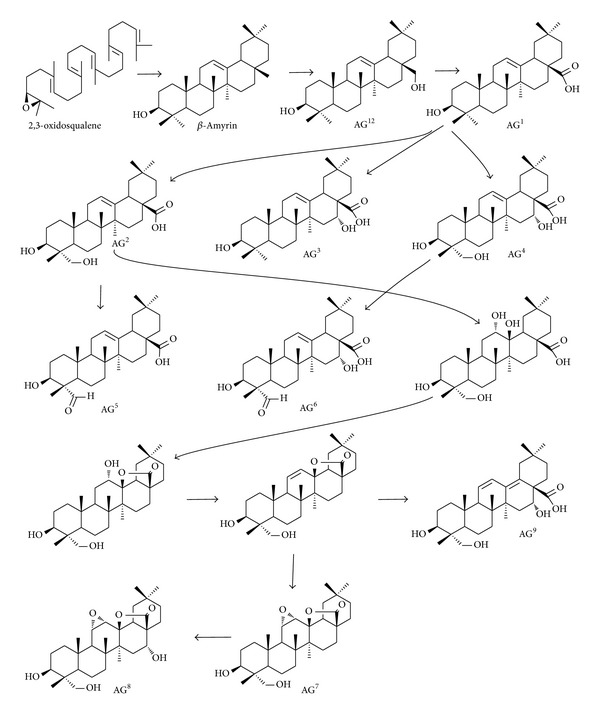
Hypothesized biosynthetic pathway for oleanane aglycones from genus* Caulophyllum*. A series of step-by-step actions from 2,3-oxidosqualene to *β*-amyrin, erythrodiol, oleanolic acid, and other aglycones are assumed.

Twelve kinds of aglycones have been discovered from the genus* Caulophyllum* (Figure 2(a)) [20, 21, 30]. Before 2009, only four kinds of sapogenins were discovered from genus* Caulophyllum*, namely, oleanolic acid (AG^1^), hederagenin (AG^2^), echinocystic acid (AG^3^), and caulophyllogenin (AG^4^). However, Ma et al. reported** 47**–**53** with abnormal sapogenins AG^5^ to AG^11^ from blue cohosh for the first time. As follows,** 54** bearing sapogenin erythrodiol was discovered from genus* Caulophyllum* in 2012 [31]. These aglycones are closely related oxygenated pentacyclic triterpenoidal structures that can be distinguished only by the positions and numbers of the double bonds in rings C and D and oxygenation patterns in positions C-16, C-23, and C-28. A possible biosynthetic pathway of* Caulophyllum* sapogenins can be hypothesized, as shown in Scheme 2. The first is, that 2,3-oxidosqualene is cyclized to the pentacyclic oleanane-type triterpenoid backbone**β**-amyrin by plant oxidosqualene cyclases**β**-amyrin synthase [32, 33]. The**β**-amyrin experienced hydroxylation at C-28 to produce erythrodiol (AG^12^). Erythrodiol is further oxidized at the C-28 position by a single cytochrome P450 enzyme to yield oleanolic acid (AG^1^) [34]. The aglycones AG^1^–AG^9^ may be derived from the common skeleton of oleanolic acid as precursors that firstly experience selective oxidation at C-23, or C-16, or both of C-23 and C-16 to afford hederagenin (AG^2^), echinocystic acid (AG^3^), and caulophyllogenin (AG^4^), respectively. Hederagenin may selectively involve a complex process such as dehydrogenization, oxidation, lactonization, dehydration, and lactone ring hydrolysis to form diverse aglycones in genus* Caulophyllum*. Though the intermediate (3*β*,12*α*-dihydroxy-olean-28-oic acid *γ*-lactone) has been artificially synthesized from oleanolic acid (Supplementary Scheme 1 in Supplementary Material available online at http://dx.doi.org/10.1155/2014/684508) [35], this type of biosynthetic pathway in plants also needs to be further confirmed.

The according carbohydrate chains are composed mainly of arabinose, rhamnose, and glucose moieties. After acid hydrolysis, gas chromatography analysis revealed the presence of glucose, arabinose, and rhamnose through comparing with derivatives obtained by the same method of standard monosaccharides [36]. For their linkage mode of sugar moieties, it showed the presence of many linked types of sugar moieties, including terminal glucose, 1,6-linked glucose, terminal rhamnose, 1,2-linked rhamnose, terminal arabinose, 1,2-linked arabinose, 1,3-linked arabinose, and 1,2,3-linked arabinose [20, 21, 30, 36] (Figure 2(b)). Diverse aglycones, monosaccharide residues, and diverse linkage mode of sugar moieties are possible to form diverse structures of triterpene saponins from genus* Caulophyllum*.

### 2.3. Other Compounds

**Figure 3:**
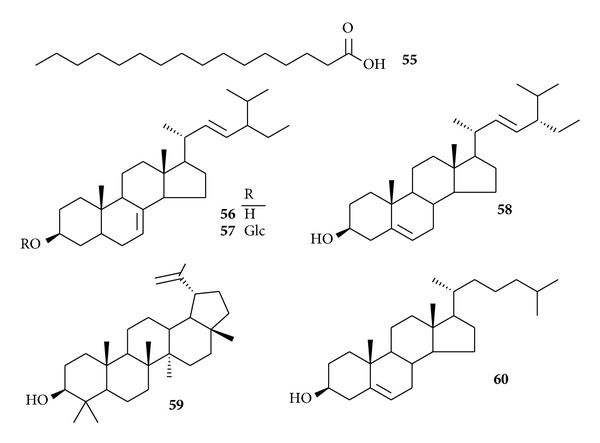
Chemical structures of other compounds (**55**–**60**) from genus* Caulophyllum*.

Other minor compounds are present in this genus, such as fatty acids and sterols [37, 38]. These compounds were identified as palmitic acid (**55**), *α*-spinasterol (**56**), *α*-spinasterol-*β*-D-glucopyranoside (**57**), stigmasterol (**58**), lupeol (**59**), and cholesterol (**60**) (Figure 3). Polysaccharides are present in this genus. The extraction process and antioxidant activity of polysaccharides have been studied [39, 40]. The optimal ultrasound-assisted extraction conditions of polysaccharides from* C. robusutm* were extraction temperature 65°C, time 70 min, ratio of liquid to solid 20, and extracting power of 70 W.

## 3. Synthetic Taspine Derivatives

**Scheme 3:**
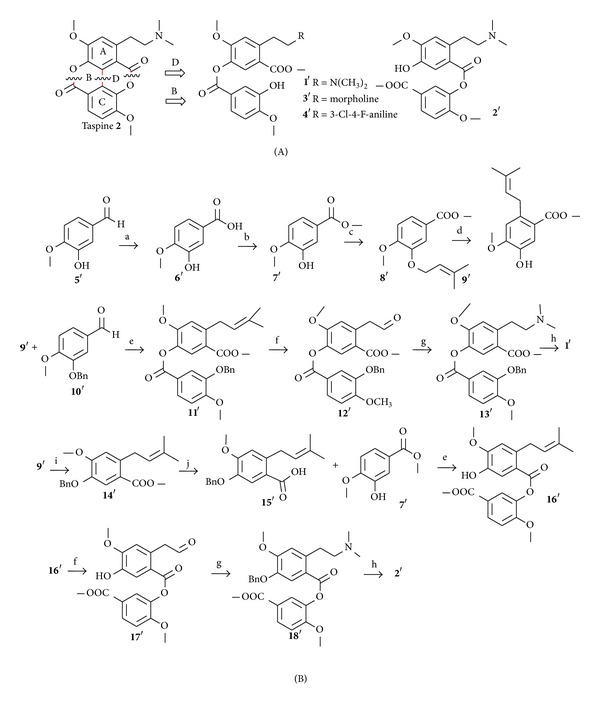
(A) Design of ring-opened target compounds 1′–4′; (B) preparation of target compounds 1′–4′. Reagents and conditions: (a) NaOH, KOH, H_2_O, 84%; (b) CH_3_OH, H_2_SO_4_, 90%; (c) prenyl bromide, K_2_CO_3_, acetone, 92%; (d) N, N-dimethylaniline, N_2_, reflux, 71%; (e) anhydrous THF, DCC, DMAP, 78%; (f) OsO_4_, NaIO_4_, acetone/H_2_O/*t*-BuOH, 32%; (g) dimethylamine /Morpholine/3-Cl-4-F-aniline, THF, CH_2_Cl_2_, NaBH(OAc)_3_, 25–40%; (h) H_2_, Pd/C, 97%; (i) BnCl, K_2_CO_3_, EtOH, 95%; (j) NaOH/H_2_O, CH_3_OH, 93% [17].

Zhang et al. designed and synthesized four novel ring-opened target compounds (1′–4′) by structure-based drug design. This design includes two pathways: cleavage of the C–C bond of diphenyl and ester bond of ring B and ring D (Scheme 3(A)). Targeted compounds 1′ and 2′ were synthesized by the route outlined in Scheme 3(B). Isovanillin (5′) was used as the starting material, which was firstly oxidized to afford isovanillic acid (6′). The methyl ester 7′ was prepared to avoid side-reactions of the carboxylate group. Then refluxing of 7′ with prenylbromide in the presence of K_2_CO_3_ in anhydrous acetone afforded 8′. A solution of 8′ in N,N-dimethylaniline was heated to reflux to give 9′. Prenyl group was moved into the paraposition of hydroxyl in Claisen rearrangement process. The next step was the coupling of 9′ to the carboxyl of 10′ by an ester bond with DCC and DMAP. The oxidation of 11′ produced aldehyde 12′, which reacted with dimethylamine followed by reduction to give 13′. At last, benzyl deprotection of 13′ with palladium-carbon in MeOH gave 1′. The 3′ and 4′ were synthesized in the same way from isovanillin [17].

**Scheme 4:**
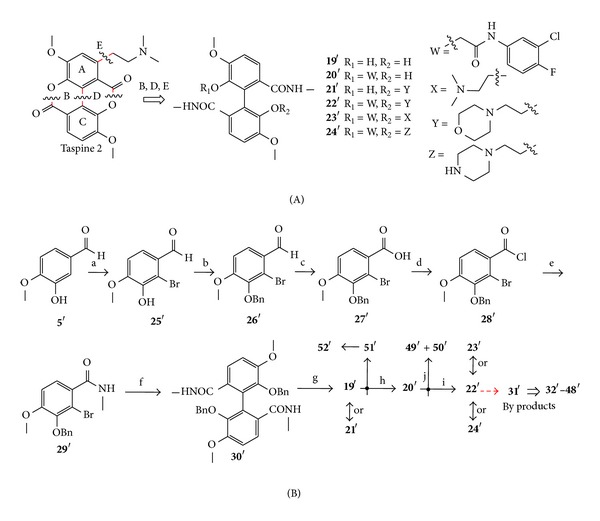
(A) Design of ring-opened target compounds 19′–24′; (B) preparation of target compounds (19′–24′). Reagents and Conditions: (a) Fe, NaOAc, AcOH, Br_2_, 81%; (b) BnCl, K_2_CO_3_, 95%; (c) NaH_2_PO_4_, NaClO_2_, 30% H_2_O_2_, 93%; (d) SOCl_2_, DMF(cat), CH_2_Cl_2_, 96%; (e) CH_2_Cl_2_, 30% CH_3_NH_2_, 85%; (f) Cu, DMF, 72%; (g) H_2_, Pd/C; 97%; (h) K_2_CO_3_, DMF, (26, 64%; 25, 76%); (i) K_2_CO_3_, EtOH, (27, 72%; 28, 59%; 29, 54%) [18, 42–44].

**Figure 4:**
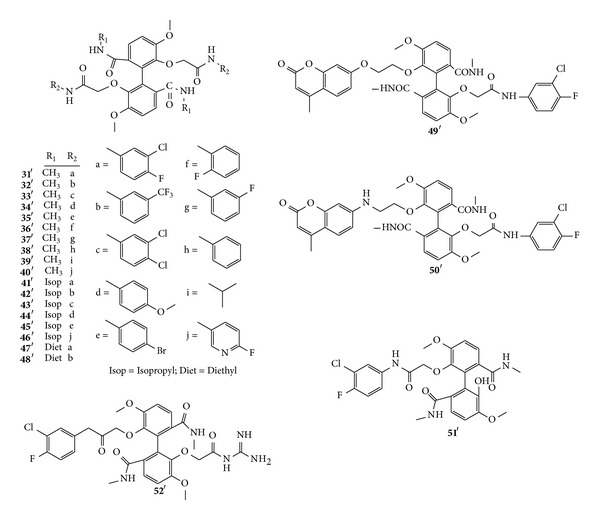
Structures of 31′–51′.

Synthetic endeavors into cleavage of the C–C bond and ester bond of rings B, D, and E have been studied (Scheme 4(A)). Initially, six target biphenyl derivatives (19′–24′) were successfully synthesized by general routes described in Scheme 4(B) employing a classical symmetrical Ullmann reaction [18]. Isovanillin (5′) was also used for the starting material, which was required for seven steps to afford 19′ by bromination, benzylation, oxidation, substitution reaction, Ullmann reaction, and catalytic hydrogenation. 19′ is an important intermediate to synthesize the following targeted compounds. During the synthesis of unsymmetrical biphenyl (22′), a novel symmetrical biphenyl derivative (31′) was surprisingly isolated as a byproduct [41], which exhibited potent anticancer activity to attract increasing attention. To further investigate this finding, researchers aimed to enhance the structural complexity and diversity of 22′ by generating novel biphenyls (Figure 4) [41]. As a result, eighteen symmetrical biphenyls derivatives (31′–48′) were firstly prepared [42]. Following these, He et al. used 20′ as the identifying group and synthesized another two novel taspine diphenyl derivatives 49′ and 50′, which were made by introducing coumarin groups into the structure of 20′ [43]. Meanwhile, derivatives 51′ and 52′ were obtained via similar procedures (Scheme 4(B)) [44].

## 4. Bioactivity

### 4.1. Antibacterial Activity

Earlier biological studies showed that caulosides A–D and G (**26**,** 40**,** 27**–**29**) have antimicrobial activity [45]. Recently, triterpene compounds isolated from* C. robustum* showed microorganism inhibitory activities to the test fungi and bacteria. Moreover, compound** 35** and cauloside B (**40**) had notable inhibiting microorganism activities to bacteria with minimal inhibitory concentration (MIC) of 3.9 *μ*g/mL [11]. Ethanol extract and its five subfractions of* C. robustum* showed high antibacterial activity against* Staphylococcus aureus*,* Staphylococcus aureus* (clinic bacterial), and* Bacillus subtilis*, and the diameters of the biggest inhibition zone were 20.03 mm, 23.52 mm, and 20.77 mm. The MICs of these were 0.31–0.63 mg/mL [46].

### 4.2. Anti-Inflammatory and Analgesic Effects

The anti-inflammatory and analgesic effects of ethanol extract, chloroform extract, and* n*-butyl alcohol extract from* C. robustum* were observed by several animal experiments. Among the different organic extracts, the action of alcohol extract was better than other organic extracts [12]. Cauloside A (**26**) and cauloside C (**27**) had anti-inflammatory and analgesic activities at dose dependency and the analgesic effect was the most significant when compounds were injected for 30 min [11]. From the points of structure-activity relationship of the saponins, cauloside C (**27**) with disaccharide has more potent analgesic effect than cauloside A (**26**) with monosaccharide. Oppositely, cauloside A (**26**) has more potent anti-inflammatory activity than cauloside C (**27**). The anti-inflammatory activity of taspine hydrochloride has been demonstrated by using the carrageenan-induced pedal edema method, the cotton pellet-induced granuloma method, and the adjuvant polyarthritis model [47].

Lee et al. (2012) assessed the* in vitro* and* in vivo* effects of blue cohosh on lipopolysaccharide (LPS)-induced cytokines in BV2 cells and mice. Several lines of evidence indicate that blue cohosh treatment suppressed the elevation of LPS-induced iNOS (inducible nitric oxide synthase) expression in a concentration-dependent manner in microglia cells. Blue cohosh saponins (caulosides A−D:** 26**,** 40**,** 27**,** 28**) significantly suppressed the expression of tumor necrosis factor-*α* (TNF-*α*), interleukin-1*β* (IL-1*β*), and IL-6. In addition, blue cohosh extract suppressed the expression of COX (cyclooxygenase)-2, iNOS, and proinflammatory cytokines in adrenal glands of mice. So, it is concluded that saponin constituents of blue cohosh exert anti-inflammatory effects through the inhibition of expression of iNOS and proinflammatory cytokines [48].

### 4.3. Antioxidant Effects

Caulophylline A−D (**18**–**21**) afforded the lower scavenging effects against DPPH (1,1-diphenyl-2-picrylhydrazyl) radical at test concentration (6.0 to 107.4 *μ*g/mL). Caulophylline E (**22**) showed good scavenging effects against DPPH radical with IC_50_ (half-inhibition concentration) of 12.1 *μ*g/mL [13]. Antioxidant activities of the polysaccharide fraction, ethanol extract, and different polar fractions of* C*.* robustum* were evaluated by DPPH, hydroxyl, and superoxide radical and nitrogen dioxide (NO_2_) scavenging assay [39, 49]. The results showed that ethanol extract and different polar fractions displayed high antioxidant activities. The scavenging activities of polysaccharides from* C. robustum* for DPPH, hydroxyl, and superoxide radical and NO_2_ were attributed to 80%, 96%, 78%, and 85.1%, respectively, for the concentrations of 5.0 mg/mL. Another experiment research reported that chloroform partition fraction showed IC_50_ value of DPPH-free radical-scavenging activity which was 79.4 *μ*g/mL [14].

### 4.4. Antiacetylcholinesterase Activity

As early as 2006, taspine (**2**) has been confirmed to be an antiacetylcholinesterase (AChE) inhibitory agent by a bioactivity-guided approach in a* Magnolia x soulangiana* extract using a microplate enzyme assay with Ellman's reagent [50].** 2** showed a significantly higher effect on AChE than the positive control galantamine and selectively inhibited the enzyme in a long-lasting and concentration-dependent fashion with an IC_50_ value of 0.12 *μ*g/mL. It could be suggested that taspine might be a potential candidate for the development of anti-AD (Alzheimer's disease) treatment.

More recently,* C. robustum* has been confirmed to possess significant AChE activity with inhibition rates (88.72 ± 1.47)% at the concentration of 1 g·L^−1^ through thin layer chromatography bioautographic method. Furthermore, chloroform fractions have shown higher AChE inhibitory capacity, so it will be further performed bioguided isolation and purification to obtain active compounds [14]. In addition to taspine in* C*.* robustum*, whether to have other compounds responsible for the activity of AChE is worthy of studying further.

### 4.5. Effect on Atherosclerosis and Myocardial Ischemia

It was found that the* n*-butanol fraction of* C*.* robustum* was an effective part, and caulophine (**17**) separated from the part was an active one in vasodilatation [51, 52]. The* n*-butanol fraction may have protective action on H_2_O_2_ injured-human umbilical vein endothelial cell line* in vitro,* and its mechanism of action may be related to the increase of the level of nitric oxide (NO), NOS (nitric oxide synthase), and the expression of NF-*κ*B (nuclear factor kappa B) [53]. The interaction between the effective component** 17** and the membrane or membrane receptor was reflected in the vascular CMC model, which suggested that** 17** may exert bioactivity in the heart [52]. The deeper study demonstrates that** 17** is able to protect cardiomyocytes from oxidative and ischemic injury through an antioxidative mechanism [54] and from caffeine-induced injury via calcium antagonism [51].

### 4.6. Antitumor Activity and Mechanism of Action

The cytotoxicity (IC_50_) of taspine (**2**) was found to be 0.39 *μ*g/mL against KB cells and 0.17 *μ*g/mL against V-79 cells [55].** 2** showed antitumor activity on the mouse S180 sarcoma in a good dose-dependent manner [56]. The inhibition rates on tumor of taspine at low, middle, and high concentrations were 39.08%, 43.99%, and 48.60%, respectively. The microvessel density and protein expressing of the vascular endothelial growth factor (VEGF), basic fibroblast growth factor (bFGF), Bcl-2, and Bax in the tumor were decreased compared with the negative control. The ratio of Bax to Bcl-2 was increased.** 2** has antitumor effect on the S180 sarcoma, and the mechanism may be through the way of decreasing the expressing of the VEGF, bFGF, Bcl-2, and Bax and inducing the vascular endothelial cell apoptosis.

Zhan et al. was to investigate the effect of taspine on the growth of oestrogen-receptor-positive breast cancer xenografts* in vivo* and the possible mechanism for this action [19]. Cell cycle and apoptosis analysis documented that taspine was able to change cell cycle and induce cell apoptosis. There was a significant decrease in the expression of estrogen receptor (ER) and progesterone receptor (PR) both in tumor tissue and cells after treatment with taspine. At the same time, it also showed a reduction in the expression of mRNA for ER and PR in the group treated with taspine. These data suggested that taspine might serve as a promising candidate of ER antagonist in the treatment of oestrogen-independent breast cancer.

Many evidences have shown that taspine could suppress tumor-induced angiogenesis. Taspine was able to inhibit chicken chorioallantoic membrane angiogenesis through interfering with the proliferation and migration of endothelial cells in a dose-dependent manner [57]. The exact mechanism [15] has been further demonstrated, suggesting that VEGF and bFGF secretion were downregulated by taspine in human non-small cell lung cancer cell (A549 cell) and human umbilical vein endothelial cells (HUVECs), confirmed by the decreased mRNA level of VEGF and Flk-1/KDR after taspine treatment in HUVECs. The molecular mechanisms of taspine on tumor angiogenic inhibition have been further studied* in vitro* [58], which indicated that taspine significantly inhibited cell proliferation of HUVECs induced by VEGF165 via decreasing Akt and Erk1/2 activities except decreasing VEGF level. Authors assume that taspine can inhibit the proliferation of vascular endothelial cells in tumor by regulating PI3 kinase and MAP kinase signal pathways.

Additionally, taspine could induce apoptosis of HUVECs in a dose-dependent manner [59]. Cell cycle was significantly stopped at the S phase. The morphology of HUVEC treated with taspine showed nuclear karyopycnosis, chromatin agglutination, and typical apoptotic body detected by electronic microscope. Taspine has an inhibitory effect on growth of HUVECs and can induce its apoptosis by decreasing Bcl-2 expression and increasing bax expression. Zhang et al. continuously investigated the effects of taspine on the proliferation and apoptosis in the A431 cell [60]. The cell cycle was significantly stopped at S phase, and nuclear karyopyknosis, chromatin agglutination, and typical apoptotic bodies were found after taspine treatment in A431 cells. There was a decrease in the expression of Bcl-2, whereas the expression of caspase-3, cleaved caspase-3, CDK2, CDK4, and Bax increased. These data demonstrated that taspine can induce apoptosis by activating caspase-3 expression and upregulating the ratio of Bax/Bcl-2 in A431 cells.

The preliminary biological test demonstrated that derivative 3′ showed much better inhibitory activities against CACO-2 (IC_50_ = 0.023 *μ*g/mL) and ECV304 (IC_50_ = 0.0012 *μ*g/mL) than taspine [17]. A deep research demonstrated that most derivatives (1′–4′ and 19′–24′) possessed a moderate degree of cytotoxicity against human cancer cell lines [18]. One of them (3′) exhibited much better antiproliferative activity against CACO-2 (IC_50_ = 23.4 *μ*g/mL) and ECV304 (IC_50_ = 1.19 *μ*g/mL) cells than taspine did. Some of the compounds showed good antiproliferative activity against colon (HT29), breast (MCF-7), lung (A549), rectum (CACO-2), skin (A375), hepatoma (7721), and pancreatic (PANC-1) cancers cell lines. A continual research demonstrated that derivative 3′ can inhibit the proliferation of, and induce apoptosis in, Caco-2 cells by activating caspase-3, caspase-8, and caspase-9, downregulating the expressions of VEGF, and upregulating the ratio of bax/bcl-2 [61].

Derivatives (31′) and (32′) demonstrated the most potent cytotoxic activity with IC_50_ values between 14.2 *μ*g/mL and 22.3 *μ*g/mL among symmetrical taspine derivatives (31′–48′) [42]. Biphenyls without halogen substitution (34′, 38′, 39′, and 44′) were much less potent than those containing halogen. Halogen substitution played a critical role in the activity of biphenyls. Derivative 31′ inhibits tumor growth in xenografted A549 cells in nude mice by inhibiting the growth of neovessels. In other words, derivative 31′ is an inhibitor of angiogenesis which functions by downregulating VEGF [62]. Furthermore, derivative 31′ had potential to suppress the adhesion, migration, and invasion of ZR-75-30 cancer cells, and it could serve as a potential novel therapeutic candidate for the treatment of metastatic breast cancer [63]. Derivative 48′ could inhibit proliferation of lovo cell and tumor growth in a human colon tumor xenografted model of athymic mice, which might be a novel angiogenesis inhibitor that reduces angiogenic responses* in vivo* and* in vitro* by blocking VEGFR signaling pathways [64].

Two novel derivatives (49′ and 50′) by introducing different coumarin fluorescent groups into the basic structure have not only fluorescence but also the ability to inhibit effects on different breast cancer cell lines, which indicates their possible further use as dual functional fluorescence probes in tracer analysis. Derivative 51′ inhibits tumor growth and cell proliferation by inhibiting cell migration, downregulating mRNA expression of VEGF and EGF, and decreasing angiogenic factor production, which deserves further consideration as a chemotherapeutic agent [44]. All evidences have demonstrated that the lactone ring B is important for activity, while the lactone ring D can be opened, thus retaining and even improving the antiproliferative properties of taspine. Halogen substitution could potentially improve the anticancer activity of the biphenyl derivatives [17, 18, 42].

### 4.7. Inhibitory Cytochrome P450 Effects

The methanolic extracts of the roots of blue cohosh, the alkaloidal fraction, and isolated constituents were evaluated for their inhibition of major drug metabolizing cytochrome P450 (CYP450) enzymes [65]. The methanolic extracts did not show any effect but the alkaloidal fraction showed a strong inhibition of CYP2C19, 3A4, 2D6, and 1A2 (>80% inhibition at 100 *μ*g/mL) with IC_50_ values in the range of 2–20 *μ*g/mL. Among the isolated alkaloids, caulophyllumine B (**15**), O-acetlybaptifolin (**11**), anagyrine (**4**), and lupanine (**9**) inhibited these enzymes to various extents (IC_50_: 0.5–15.1 *μ*g/mL). N-methylcytisine (**6**) showed weak activity against the CYP3A4* in vitro* with 32% inhibition at 20.4 *μ*g/mL. An equimolar mixture of alkaloids exhibited a more pronounced inhibitory effect on all four enzymes as compared to the isolated alkaloids. Among the saponins, caulosides C (**27**) and D (**28**) showed 43% and 35% inhibition of CYP3A4 at the concentration of 76.6 and 107.4 *μ*g/mL, respectively. Other enzymes were not affected. This* in vitro* study indicates that dietary supplements containing blue cohosh may pose a risk of drug-drug interactions if taken with other drugs or herbs, metabolism of which involves CYP450 enzymes.

### 4.8. Topoisomerase Inhibitor

Taspine (**2**) was found to induce conformational activation of the proapoptotic proteins Bak and Bax, mitochondrial cytochrome c release, and mitochondrial membrane permeabilization in HCT116 cells [66]. Analysis of the gene expression signature of taspine treated cells suggested that taspine is a topoisomerase inhibitor. Taspine has a reduced cytotoxic effect on a cell line with a mutated topoisomerase II enzyme. Interestingly, in contrast to the topoisomerase II inhibitors doxorubicin, etoposide, and mitoxantrone, taspine was cytotoxic to cell lines overexpressing the PgP or MRP drug efflux transporters. Taspine induces wide-spread apoptosis in colon carcinoma multicellular spheroids and that apoptosis is induced in two xenograft mouse models* in vivo*. Taspine is a dual topoisomerase inhibitor that is effective in cells overexpressing drug efflux transporters and induces wide-spread apoptosis in multicellular spheroids.

### 4.9. Effect on Wound Healing

A patent reported that the method is useful for preparing wound care composition, which comprises* C. robustum*, which is useful for relieving postoperative pain and promoting wound healing and blood circulation in wound area [67]. Further research showed that taspine was able to promote early phases of wound healing in a dose-dependent manner with no substantial modification thereafter. Its mechanism of action is probably related to its chemotactic properties on fibroblasts and is not mediated by changes in extracellular matrix [68]. Authors summarized that taspine opens a pathway of research for new tools to stimulate wound repair in the absence of macrophages, thereby helping to better understand the process of wound healing. Taspine also exhibited a dose-related cicatrizant effect and a median effective dose (ED_50_) of 0.375 mg/kg, which was nontoxic to human foreskin fibroblasts at concentrations below 150 ng/mL and that had no effect on cell proliferation [69].

### 4.10. Toxicity

N-methylcytisine (**6**) exhibited teratogenic activity in the rat embryo culture (REC), an* in vitro* method to detect potential teratogens. Anagyrine (**4**) and *α*-isolupanine (**12**) were not teratogenic in the REC at tested concentrations. Taspine (**2**) showed high embryotoxicity, but no teratogenic activity, in the REC [25]. Wu et al. have observed that blue cohosh interrupted medaka embryogenesis and produced an abnormal phenotype, which identifies blue cohosh as a potent teratogen. Moreover, the induction of gata2 mRNA followed by edn1 mRNA by BC indicates that the teratogenic response of blue cohosh is probably mediated by the Gata2-End1 signaling pathway [9]. Caulosides B (**40**) and C (**21**) were reported to have cytotoxicity to developing sea urchin embryos by changing cell permeability. It is well-known that cytotoxic glycoside causes a disturbance of cell membrane permeability that can cause leakage of important cellular components [70, 71].

A new born infant whose mother ingested an herbal medication, blue cohosh, to promote uterine contractions presented with acute myocardial infarction associated with profound congestive heart failure and shock [7]. One year later, other similar cases were reported [72]. Meanwhile, According to a survey of midwives in the United States, approximately 64% of midwives reported using blue cohosh as a labour-inducing aid. Severe multiorgan hypoxic injury may occur. Recently, a review focused on the toxicity of blue cohosh has been reported [2].

## 5. Pharmacokinetics

Magnoflorine (**1**), taspine (**2**), and caulophine (**17**) were the main components of genus* Caulophyllum*. Several studies have been carried out to understand the distribution, absorption, metabolism, and excretion of magnoflorine (**1**), taspine (**2**), and caulophine (**17**) using modern analytical methods.

### 5.1. Pharmacokinetics of Magnoflorine

As far as magnoflorine is concerned, a new sample-preparation method based on hollow-fiber liquid-phase microextraction (HFLPME) was developed and successfully used for pharmacokinetic studies of magnoflorine in rat plasma after intravenous administration. The magnoflorine disappears from rat plasma in accordance with a two-compartment open model. The plasma concentration of magnoflorine reached a peak immediately after completion of administration, then began to decline. Without doubt, the chromatographic and HFLPME sample-preparation procedures of magnoflorine will facilitate the development and validation of other methods of analysis of magnoflorine in other biological matrixes [73].

### 5.2. Pharmacokinetics of Taspine

Lu et al. (2008) prepared taspine solid lipid nanoparticles (**2**-SLN) and taspine solid lipid nanoparticles with galactoside (**2**-G2SLN) separately using the film evaporation extrusion method. The pharmacokinetics and liver target efficiency after IV administrations of** 2**-SLN and** 2**-G2SLN to ICR mice were finally compared [59]. The pharmacokinetics and tissue distribution after intravenous administrations of taspine solution and taspine liposome to ICR mice were compared. Incorporation into liposomes prolonged taspine retention within the systemic circulation and increased its distribution to the spleen and liver but reduced its distribution to the heart and brain [74].

### 5.3. Pharmacokinetics of Caulophine

Pharmacokinetic studies have shown that caulophine (**17**) is easily absorbed after oral administration, but it is eliminated from the body slowly. In fact, 1.25 h after treating rats treated with caulophine, the highest concentration of caulophine was found in the liver. Therefore, hepatic metabolism is probably the main route for the* in vivo* processing of caulophine [75]. Two metabolites including glucuronide conjugate and N-oxide of caulophine were found in rat urine and feces by HPLC-MS. Moreover, the same caulophine glucuronide conjugate was observed in rat liver microsomes system. However, caulophine glucuronide conjugate was not observed in dog liver microsomes [28].

## 6. Cell Membrane Chromatography for Activity Screening

Cell membrane chromatography (CMC) is a novel bioaffinity chromatographic technique. The CMC combined with high performance liquid chromatography (HPLC) or HPLC/MS will be of great utility in drug discovery using natural medicinal herbs as a source of novel compounds. In reported studies, the model of CMC in which cell membrane is enriched with certain receptors is used, as the stationary phase was applied to screen the target components from medicinal herbs [76–78] and to investigate the interactions between drug and receptor [79, 80]. This system has been successfully applied to the screening and identification of active components from* C*.* robustum*.

A combined A431/CMC-HPLC method was developed and was successfully applied to recognize, separate, and identify target components “taspine” and “caulophine” from* C*.* robustum* [81]. A combined A431/CMC with online HPLC/MS was also established for identifying active components from* C*.* robustum* acting on human epidermal growth factor receptor (EGFR) [77]. Retention fractions on A431/CMC model were captured onto an enrichment column and the components were directly analyzed by combining a 10-port column switcher with an LC/MS system for separation and preliminary identification. Using sorafenib tosylate as a positive control, taspine (**2**) and caulophine (**17**) were identified as the active molecules which could act on the EGFR. Other research results showed that taspine (**2**) was the active molecule acting on the tumor vasodilatation [52], and magnoflorine (**1**) and caulophine (**17**) were the active molecules acting on the human *α*_1A_-adrenoceptor (*α*_1A_AR) [82].

This system has been also successfully applied to investigate the interactions between active compounds from* C*.* robustum* and receptor. A new high-expression vascular endothelial growth factor receptor-2 (VEGFR-2) CMC method combined with mathematical treatments was proposed for evaluating taspine-receptor interactions [83]. A competitive binding study was performed and the results indicate that there are multiple types of binding sites on VEGFR-2 for taspine (**2**). Following this, Du and coworkers developed another new high-expression EGFR CMC method to recognize the ligands acting on EGFR specifically and investigate the affinity of gefitinib/a novel taspine derivative HMQ1611 to EGFR [84]. It has been proven that the CMC method combined zonal elution provides a powerful technique for the characterization of HMQ1611 binding to the EGFR.

## 7. Conclusions and Future Prospects

The present review discusses the chemistry and pharmacological aspects of the genus* Caulophyllum* and especially provides a detailed analysis of the literature published since the year of 2000. The state of the science on* Caulophyllum* chemistry and pharmacological activity leaves considerable opportunity for future discoveries.

Two new classes of alkaloids, piperidine-acetophenone conjugates (**13**–**16**) and fluorenone (**17**–**22**) alkaloids, have been reported from genus* Caulophyllum*, suggesting that piperidine-acetophenone conjugates and fluorenone type alkaloids are another two major kinds of metabolites that existed in this genus* Caulophyllum.* In addition to common aglycones (oleanolic acid, hederagenin, echinocystic acid, and caulophyllogenin), eight other kinds of aglycones have been found from* Caulophyllum* species. Diverse aglycones, monosaccharide residues, and linked modes of sugars are possible to form diverse structures of triterpene saponins from genus* Caulophyllum*. Many new compounds have been identified in recent years, and we are convinced that more trace constituents with novel structures will be discovered with the development of new technology for isolation and identification.

Currently, although many purified compounds have been tested for activity which are** 1**,** 2**,** 4**,** 5**,** 6**,** 9**,** 11**,** 15**,** 17**,** 26**–**29**,** 35,** and** 40**, only** 2** (taspine) is performed in-depth study on its anti-tumor and anti-angiogenic mechanisms and could serve as a lead compound in anticancer agent development. Meanwhile, a class of biphenyl derivatives of taspine was designed and synthesized for screening potential novel anticancer agents. Besides** 2**, caulophine (**17**) was identified as another active molecule which could act on the EGFR and *α*_1A_AR by combining *α*_1A_AR/CMC and A431/CMC with online HPLC/MS.** 17** also merits further research to see its action of mechanisms. On the other hand, a number of compounds with novel structure skeleton, such as** 13**–**16**,** 18**–**22**,** 50**,** 51**,** 52**,** 53**, and** 54** have previously been isolated, but no further tests have been performed. It is possible that these compounds are usually overlooked due to their low abundance in* Caulophyllum*. Pharmacokinetic study is also limited for compounds isolated, mainly involving three active alkaloids** 1**,** 2,** and** 17**. So it is very urgent to develop pharmacokinetic study* in vivo* for other bioactive compounds in genus* Caulophyllum*.

Pharmacological studies carried out on crude extracts and pure metabolites provided pragmatic documents for its traditional uses and have revealed that this genus is a valuable source for medicinally important molecules. Many important biological activities of this genus have been demonstrated such as anti-inflammatory and analgesic effects, antioxidant effects, antiacetylcholinesterase activity, and antitumor et al. Though many promising results were confirmed by animal models, it should be further investigated by clinical trials. Regarding the constituents contributed to medicinal values, the findings indicated that alkaloids and triterpene saponins were regarded as the major constituents in this genus, while polysaccharides that occurred in the genus are worthy of further researching their chemical and pharmacological activities [37]. However, most of the plant extracts used in the above bioassay were not well characterized, and this defect led to the difficulty to reproduce the reported results. To add the availability of primary experimental data, suitable analytical and standardization protocols of plant materials should be developed, since these are the ground work for convincing and reproducible pharmacological studies.

The toxicity of* Caulophyllum* species is not negligible, mainly involving the teratogenic effects and inducing heart failure and shock by ingesting blue cohosh. From the view of current research results, alkaloid fractions may be responsible for major toxicity. However, exact individuals are required for further research by chemical and pharmacological experiments. The future work should be focused on the relationship between clinical effects and side-effects of* Caulophyllum* extracts to screen a safe and effective dosage. Moreover, a strict quality control procedure should be adopted to guarantee its quality. On the other hand, the alkaloids and triterpene saponins are two major kinds of constituents in blue cohosh, which are easily divided by chromatography methods [22]. The individual pharmacological tests for fractions of alkaloids and triterpene saponins should be considered according to the traditional and modern uses of* Caulophyllum* plants. Whether alkaloids and triterpene fractions can be used separately in the future according to each medical function, it may be a good choice for* Caulophyllum* plants for reducing the drug interaction and enhancing their efficiency.

## Supplementary Material

Chemical synthesis of 3*β*,12*α*-dihydroxy-olean-28-oic acid *γ*-lactone from oleanolic acid. Reagents and conditions: O_3_/CHCl_3_:MeOH/-78 C/30 min (70%)

## Figures and Tables

**Table 1:** Chemical structures of alkaloids (**1**–**22**) from genus* Caulophyllum*.

No.	Compounds	Formula	Sources^a^	References
**1**	Magnoflorine	C_20_H_24_NO_4_^+^	*Cr, Ct *	[85, 86]
**2**	Taspine	C_20_H_19_NO_6_	*Cr, Ct *	[25, 86]
**3**	Boldine	C_19_H_21_NO_4_	*Cr *	[86]
**4**	Anagyrine	C_15_H_20_N_2_O	*Cr, Ct *	[20, 85, 86]
**5**	Sparteine	C_15_H_26_N_2_	*Cr, Ct *	[25]
**6**	N-methylcytisine	C_12_H_16_N_2_O	*Cr, Ct *	[20, 85–87]
**7**	Cytisine	C_11_H_14_N_2_O	*Cr *	[86]
**8**	5,6-Dehydro-*α*-isolupanine	C_15_H_22_N_2_O	*Cr, Ct *	[25, 86]
**9**	Lupanine	C_15_H_24_N_2_O	*Cr, Ct *	[25, 86]
**10**	Baptifoline or Argentamin	C_15_H_20_N_2_O_2_	*Ct *	[85, 86]
**11**	O-acetylbaptifolin	C_17_H_22_N_2_O_3_	*Ct *	[20]
**12**	*α*-isolupanine	C_15_H_24_N_2_O	*Cr, Ct *	[25, 86]
**13**	Thalictroidine	C_14_H_19_NO_2_	*Ct *	[20, 25]
**14**	Caulophyllumine A	C_15_H_21_NO_4_	*Ct *	[20]
**15**	Caulophyllumine B	C_14_H_19_NO	*Ct *	[20]
**16**	Piperidylacetophenone	C_15_H_21_NO_3_	*Ct *	[20]
**17**	Caulophine	C_19_H_21_NO_5_	*Cr *	[88]
**18**	Caulophylline A	C_20_H_23_NO_5_	*Cr *	[13]
**19**	Caulophylline B	C_19_H_21_NO_5_	*Cr *	[13]
**20**	Caulophylline C	C_20_H_24_NO_5_	*Cr *	[13]
**21**	Caulophylline D	C_20_H_24_NO_6_	*Cr *	[13]
**22**	Caulophylline E	C_18_H_17_NO_4_	*Cr *	[13]

^a^ *Cr* means *C. robustum*; *Ct* means *C. thalictroides*.

**Table 2:** Chemical structures of triterpene saponins (**23**–**54**) from genus* Caulophyllum*.

No.	Compound names	C-3	C-28	Formula	Sources^a^	References
**23**	Ara→3**β**-*O*-AG^1^	S_1_	—	C_35_H_56_O_7_	*Ct *	[21]
**24**	Saponin PE, Glc → ^2^Ara → 3**β**-*O*-AG^1^	S_2_	—	C_41_H_66_O_12_	*Ct *	[20, 21]
**25**	Ciwujianoside A, Glc → ^2^Ara → 3**β**-*O*-AG^1^-28-*O←* Glc^6 ^ *←* Glc^4^ *←* Rha	S_2_	S_3_	C_59_H_96_O_26_	*Ct *	[20, 21]
**26**	Cauloside A, Ara → 3**β**-*O*-AG^2^	S_1_	—	C_35_H_56_O_8_	*Cr, Ct *	[20, 21, 30, 31, 37, 89]
**27**	Cauloside C, Glc → ^2^Ara → 3**β**-*O*-AG^2^	S_2_	—	C_41_H_66_O_13_	*Cr, Ct *	[20, 21, 31, 37, 89]
**28**	Cauloside D, Ara → 3**β**-*O*-AG^2^-28-*O←* Glc^6^ *←* Glc^4^ *←* Rha	S_1_	S_3_	C_53_H_86_O_22_	*Cr, Ct *	[20–22, 30, 90]
**29**	Cauloside G, Glc → ^2^Ara → 3**β**-*O*-AG^2^-28-*O←* Glc^6^ *←* Glc^4^ *←* Rha	S_2_	S_3_	C_59_H_96_O_27_	*Cr, Ct *	[20–22, 30, 91]
**30**	Cauloside b, Rha → ^2^Ara → 3**β**-*O*-AG^2^	S_4_	—	C_41_H_66_O_12_	*Cr *	[92]
**31**	Cauloside c, Ara → ^3^Rha → ^2^Ara → 3**β**-*O*-AG^2^	S_5_	—	C_46_H_74_O_16_	*Cr *	[92]
**32**	AG^2^-28-*O←* Glc^6^ *←* Glc^4^ *←* Rha	—	S_3_	C_48_H_78_O_18_	*Ct *	[21]
**33**	Glc → ^3^Ara → 3**β**-*O*-AG^2^-28-*O←* Glc^6^ *←* Glc^4^ *←* Rha	S_6_	S_3_	C_59_H_96_O_27_	*Cr *	[22, 93]
**34**	Ara → 3**β**-*O*-AG^3^-28-*O←* Glc^6^ *←* Glc^4^ *←* Rha	S_1_	S_3_	C_53_H_86_O_22_	*Ct *	[30]
**35**	Ara → 3**β**-*O*-AG^3^	S_1_	—	C_35_H_56_O_8_	*Ct, Cr *	[21, 31, 37]
**36**	Glc → ^2^Ara → 3**β**-*O*-AG^3^	S_2_	—	C_41_H_66_O_13_	*Ct *	[21, 30]
**37**	Leiyemudanoside C, Glc → ^3^Ara → 3**β**-*O*-AG^3^- 28-*O ←* Glc^6^ *←* Glc^4^ *←* Rha	S_6_	S_3_	C_59_H_96_O_27_	*Cr *	[36]
**38**	Ara → 3**β**-*O*-AG^3^-28-*O←* Glc^6^ *←* Glc	S_1_	S_7_	C_47_H_76_O_19_	*Ct *	[21]
**39**	Glc → ^2^Ara → 3**β**-*O*-AG^3^-28-*O←* Glc^6^ *←* Glc^4^ *←* Rha	S_2_	S_3_	C_59_H_96_O_27_	*Cr *	[30]
**40**	Cauloside B, Ara → 3**β**-*O*-AG^4^	S_1_	—	C_35_H_56_O_9_	*Cr, Ct *	[20–22, 30, 31, 37, 94]
**41**	Leonticin D, Ara → 3**β**-*O*-AG^4^-28-*O←* Glc^6 ^ *←* Glc^4^ *←* Rha	S_1_	S_3_	C_53_H_86_O_23_	*Ct *	[21, 22, 30]
**42**	Cauloside H, Glc → ^2^Ara → 3**β**-*O*-AG^4^-28-*O←* Glc^6^ *←* Glc^4^ *←* Rha	S_2_	S_3_	C_59_H_96_O_28_	*Ct *	[20]
**43**	Leiyemudanoside A, Ara → 3**β**-*O*-AG^4^-28-*O←* Glc^6^ *←* Glc	S_1_	S_7_	C_47_H_76_O_19_	*Cr *	[36]
**44**	Leiyemudanoside B, Glc → ^3^Ara → 3**β**-*O*-AG^4^-28-*O←* Glc^6^ *←* Glc^4^ *←* Rha	S_6_	S_3_	C_59_H_96_O_28_	*Cr *	[36]
**45**	Glc → ^2^Ara → 3**β**-*O*-AG^4^	S_2_	—	C_41_H_66_O_14_	*Ct *	[21]
**46**	Ara → 3**β**-*O*-AG^4^-28-*O←* Glc	S_1_	S_8_	C_41_H_66_O_14_	*Ct *	[21]
**47**	Ara → 3**β**-*O*-AG^5^	S_1_	—	C_35_H_54_O_8_	*Ct *	[21]
**48**	Ara → 3**β**-*O*-AG^6^	S_1_	—	C_35_H_54_O_9_	*Ct *	[21]
**49**	Glc → ^2^Ara → 3**β**-*O*-AG^7^	S_2_	—	C_41_H_64_O_14_	*Ct *	[21]
**50**	Ara → 3**β**-*O*-AG^8^	S_1_	—	C_35_H_54_O_10_	*Ct *	[21]
**51**	Ara → 3**β**-*O*-AG^9^	S_1_	—	C_35_H_54_O_9_	*Ct *	[21]
**52**	Glc → ^2^Ara → 3**β**-*O*-AG^10^	S_2_	—	C_41_H_68_O_13_	*Ct *	[21]
**53**	Glc → ^2^Ara → 3**β**-*O*-AG^11^	S_2_	—	C_41_H_66_O_12_	*Ct *	[21]
**54**	Ara → 3**β**-*O*-AG^12^	S_1_	—	C_35_H_58_O_8_	*Cr *	[31]

^a^ *Cr* means *C. robustum*; *Ct* means *C. thalictroides*.

## Abbreviations

AChE: Antiacetylcholinesterase
AG: Aglycones
AR: Adrenoceptor
Ara: *α*-L-Arabinopyranose
Bfgf: Basic fibroblast growth factor
CMC: Cell membrane chromatography
COX: Cyclooxygenase
CYP450: Cytochrome P450
DPPH: 1,1-Diphenyl-2-picrylhydrazyl
EGFR: Epidermal growth factor receptor
ER: Estrogen receptor
GC/MS: Gas chromatography/mass spectrum
Glc: *β*-D-Glucopyranose
HFLPME: Hollow-fiber liquid-phase microextraction
HPLC: High performance liquid chromatography
HPLC/MS: High performance liquid chromatography/mass spectrum
HUVECs: Human umbilical vein endothelial cells
IC_50_: Half-inhibition concentration
IL: Interleukin
iNOS: Inducible nitric oxide synthase
LPS: Lipopolysaccharide
MIC: Minimal inhibitory concentration
NF-*κ*B: Nuclear factor kappa B
NO: Nitric oxide
NO_2_: Nitrogen dioxide
PR: Progesterone receptor
REC: Rat embryo culture
Rha: *α*-L-Rhamnopyranose
TNF-*α*: Tumor necrosis factor-*α*
TV: Tumor vasodilatation
VEGF: Vascular endothelial growth factor
VEGFR-2: Vascular endothelial growth factor receptor-2.
