# Supplementary material for: Genus Caulophyllum: An Overview of Chemistry and Bioactivity
Source: Evid Based Complement Alternat Med. 2014 May 4;2014:684508. doi: 10.1155/2014/684508 (PMC4024411; doi:10.1155/2014/684508)
Supplement: Supplementary file 1 — Chemical synthesis of 3β,12α-dihydroxy-olean-28-oic acid γ-lactone from oleanolic acid. Reagents and conditions: O3/CHCl3:MeOH/-78 C/30 min (70%) [file 684508.f1.docx]

**Supplementary Material**

**Genus** ***Caulophyllum*: An Overview of Chemistry and Bioactivity**

Yong-Gang Xia,^1^ Guo-Yu Li,^2^ Jun Liang,^1^ Bing-You Yang,^1^

Shao-Wa Lü,^1^ Hai-Xue Kuang^1^

^1^ *Key Laboratory of Chinese Materia Medica (Heilongjiang University of Chinese Medicine), Ministry of Education, Harbin, 150040, P.R. China;*

^2^ *Pharmaceutical College, Harbin Medical University, Harbin 150086,* *P.R. China*

Correspondence should be addressed to Hai-Xue Kuang; hxkuang@hotmail.com

**Supplementary Scheme 1.**
